# Growing Up with Asperger’s Syndrome: Developmental Trajectory of Autobiographical Memory

**DOI:** 10.3389/fpsyg.2012.00605

**Published:** 2013-01-11

**Authors:** Laetitia Bon, Jean-Marc Baleyte, Pascale Piolino, Béatrice Desgranges, Francis Eustache, Bérengère Guillery-Girard

**Affiliations:** ^1^U1077, INSERMCaen, France; ^2^UMR S1077, Université de Caen Basse-NormandieCaen, France; ^3^UMR S1077, École Pratique des Hautes ÉtudesCaen, France; ^4^Service de Psychiatrie de l’Enfant et de l’Adolescent, CHU de CaenCaen, France; ^5^Laboratoire Mémoire et Cognition, Institut de Psychologie, Université René DescartesBoulogne Billancourt, France; ^6^UMR S894, INSERM, Centre de Psychiatrie et Neuroscience, Université Paris DescartesParis, France; ^7^UMR S1077, CHU de CaenCaen, France

**Keywords:** autobiographical memory, autism, child, theory of mind, episodic memory, semantic memory

## Abstract

Autobiographical memory (AM) and social cognition share common properties and both are affected in autism spectrum disorders (ASD). So far, most of the scant research in ASD has concerned adults, systematically reporting impairment of the episodic component. The only study to be conducted with children concluded that they have poorer personal semantic knowledge than typical developing children. The present study explores the development of both components of AM in an 8-year-old boy diagnosed with Asperger’s syndrome, based on three examinations in 2007, 2008, and 2010. On each occasion, he underwent a general neuropsychological assessment including theory of mind (ToM) tasks, and a specially designed AM task allowing us to test both the semantic and the episodic components for three lifetime periods (current year, previous year, and earlier years). We observed difficulties in strategic retrieval and ToM, with a significant improvement between the second and third examinations. Regarding AM, different patterns of performance were noted in all three examinations: (1) relative preservation of current year personal knowledge, but impairment for the previous and earlier years, and (2) impairment of episodic memory for the current and previous year, but performances similar to those of controls for the earlier years. The first pattern can be explained by abnormal forgetting and by the semanticization mechanism, which needs verbal communication and social interaction to be efficient. The second pattern suggests that the development of episodic memory only reached the stage of “event memory.” This term refers to memory for personal events lacking in details or spatiotemporal specificity, and is usually observed in children younger than five. We conclude that the abnormal functioning of social cognition in ASD, encompassing social, and personal points of view, has an impact on both components of AM.

## Introduction

A growing body of research has demonstrated that autobiographical memory (AM) and social cognition share several common properties (Spreng and Mar, 2012). Given this functional overlap, we would expect AM to be affected in Autism Spectrum Disorders (ASD), as social cognition is impaired in this pathology. ASD includes a range of neurodevelopmental disorders including autism, Asperger’s syndrome, and pervasive developmental disorders that are characterized by abnormalities in three core areas: verbal and non-verbal communication, social interaction, and restricted interests, and stereotyped behaviors. Within this spectrum, Asperger’s syndrome differs from autism in that there is no clinically significant cognitive or language delay.

Autobiographical memory refers to personal knowledge and events relating to our own lives, and contributes to the development of social cognition, from both a social and a personal point of view. First, our memories of the past or of social interactions may help us to infer the mental states of others and allow us to adapt our own behavior accordingly. Hence, these memories help to increase our interpersonal knowledge. Second, we shape our own selves by extracting meaning from some of our autobiographical memories, described as self-defining memories, to update our self-concept and create a dynamic self-memory system (Singer, 2004). Crane et al. (2010) recently showed that ASD patients are unable to elicit meaning from their memories, which may render the self-memory relationship static, unlike that of typical adults.

Social cognition encompasses to the cognitive processes used to decode and encode the social world. It encompasses the perception of others, the self, and interpersonal knowledge (Beer and Ochsner, 2006). Theory of mind (ToM) is the social cognitive ability to impute mental states (intentions, beliefs, emotions, desires, etc.) to oneself and to others, and to use these attributions to understand, predict, and explain the behavior of oneself and others. A ToM impairment is clearly established in ASD and Baron-Cohen et al. (1985) were the first to formulate the hypothesis of a link between ToM deficit and the abnormalities in communication and social interaction displayed by individuals with ASD. Difficulties with mentalizing or ToM clearly influence episodic memory in different ways. Episodic memory relies on a dynamic sense of self as differentiated from others or the ability to consider that the self is continuous over time, from past to future, as this allows children to organize and integrate personally experienced events within their self-concepts (Povinelli et al., 1996). Because these abilities mature late in typical development, true episodic memory does not emerge until around the age of 4 or 5 years (Perner and Ruffman, 1995; Wheeler et al., 1997; Newcombe et al., 2007) and continues to develop until adolescence (Piolino et al., 2007; Willoughby et al., 2012). Converging behavioral and neuroimaging data indicate that it is the psychological or interpersonal component of the self, or higher-order psychological self-awareness that is affected in ASD (Lind, 2010; Williams, 2010; Uddin, 2011). In ASD, the restricted ToM, or more specifically “theory of own mind,” as suggested by Williams (2010), has an impact on the internal state language associated with memory narratives (Crane et al., 2010, 2011). This unstructured psychological self cannot act as an effective memory organizational system, and thus precludes individuals from recollecting specific autobiographical memories. Furthermore, the poor social skills associated with ASD limit individuals’ social interactions. These social interactions, especially parent-child interactions, play a central role in the development of AM, by allowing children to construct narratives about their personal events (Nelson and Fivush, 2004). Limited linguistic interactions may adversely affect the development of AM in ASD, and also prevent the external rehearsal of narratives which, in typical individuals, enhances memory.

The personal narratives provided in response to autobiographical questionnaires allow us to distinguish between personal semantic memory or personal knowledge, and specific or episodic memory for autobiographical events (Cermak, 1984). All of the scant research conducted in adults with ASD has reported significant difficulties, mainly concerning the episodic component (Goddard et al., 2007; Crane and Goddard, 2008; Lind and Bowler, 2010; Tanweer et al., 2010; Crane et al., 2012a). When invited to evoke memories, ASD patients provide a list of facts, rather than truly specific personal experiences (Goldman, 2008), lacking in details and with few insight terms in their narratives (Crane et al., 2010). These memories are very similar to the event memories produced by young children before the emergence of the episodic memory system, as previously suggested by Maister and Plaisted-Grant (2011). Access to AM is also impaired in ASD, according to Crane et al. (2010, 2012a). It seems to take individuals with ASD longer to retrieve specific memories than it does typical adults, and unlike the latter, self-relevance cues do not facilitate their retrieval of specific details. However, the methodologies adopted by researchers may influence performance levels. Thus, greater impairment of the episodic component has been observed when participants with ASD are given a fluency task rather than an interview task (Crane and Goddard, 2008), and they perform just as well as controls on yes-no personal life event questions that do not require them to remember any specific details (Bruck et al., 2007), or when they are asked to write down their memories-a task that limits social interaction (Crane et al., 2012b).

Episodic AM impairment can be observed in children with ASD from the age of 8 (Losh and Capps, 2003) and as early as 5 years (Bruck et al., 2007). To date, the most recent study conducted in children with ASD in this domain was published by Brown et al. (2012), and interestingly focused on the quality of event reports. When the authors analyzed memory narratives provided by a group of children with ASD aged 6–14 years, they noticed that these children included fewer emotional, cognitive (thoughts and beliefs), and perceptual terms than typical controls. Contrary to their predictions, the children with ASD produced just as many social terms as controls. However, they listed general events lacking in details and social interactions. These results are consistent with previous data published by Lee and Hobson (1998). They were interested in the social attributes of the self, as seen mainly through social interactions, and reported that children and adolescents with ASD produced fewer social self-statements than typical controls. This observation, like the smaller number of emotional and cognitive terms found in personal narratives, can be explained by the affective and cognitive ToM impairment. Given the difficulty they have understanding their own beliefs and emotions, coupled with their inability to adopt other people’s perspectives, children with ASD cannot spontaneously produce as many details as typical controls when they recall an event.

Unlike the episodic component, the semantic component seems intact in ASD adults, with no effect of lifetime period (Crane and Goddard, 2008). However, as demonstrated by Bruck et al. (2007), it does appear to be affected in children, which Lind (2010) believes reflects delayed development of the self-concept. Despite this hypothesis, there has been surprisingly little research on the development of AM in ASD, and no follow-up studies have been conducted so far, even though this approach is very useful for investigating the development of AM. First, it allows researchers to control for the interindividual variability that characterizes typical development (two individuals matched on chronological age may well be at different stages of cognitive development). This variability is even greater in ASD than in typical development, and some impairments reported in published case studies would not emerge in a group analysis. Second, longitudinal studies are a unique means of investigating how memories change over time and of testing the hypothesis of the delayed development of certain cognitive skills in ASD.

We conducted a longitudinal study of a young boy with ASD focusing on general cognitive functions and both components of AM. The purpose of this investigation was to extend our understanding of the development of personal semantic knowledge and episodic autobiographical memories by administering three examinations over the space of 4 years. Assuming that the self-memory relationship in ASD is static, rather than dynamic, owing to both limited linguistic interaction and impaired episodic AM, we predicted that Simon’s personal semantic knowledge would be strongly related to the present, or here and now, and liable to be forgotten. Concerning the episodic component, memories would presumably take the form of “event memories,” rather than truly episodic memories (Maister and Plaisted-Grant, 2011). If our assumption was correct, we would find that Simon’s earliest memories, elaborated before the age at which episodic memory normally develops, were similar to those of typical children. His more recent ones, however, encoded after the age of 6 years, would contain fewer details than those of typical children. This gap in performances between typical children and our child with ASD would increase with age, despite the therapeutic interventions.

## Materials and Methods

### Case report

We describe the neuropsychological follow-up of Simon, a young boy who was diagnosed with Asperger’s syndrome on the basis of the ICD-10 criteria and the Autism Diagnostic Interview-Revised (ADI-R; Lord et al., 1994), an experimenter-administered parent interview that yields ratings for social, communicative, and repetitive behavior symptoms based primarily on behavior reported for the 4–5 year age period. This clinical instrument was used with Simon’s mother at the age of 8 years. Three neuropsychological examinations were administered in August 2007, October 2008, and March 2010. Initial neuropsychological examination was conducted just after the diagnosis at 8 years of age, and the two others during the therapeutic follow-up.

Simon is the first-born of his family. During the first psychiatric consultation and the ADI-R interview, the mother reported that delivery, pregnancy, and motor development were all normal. Simon had no language delay: he said his first words at about 21 months and his first sentences at about 2 years. His mother started to worry when Simon, by then aged 4 years, appeared reluctant to interact socially with his peers. While he was at primary school, Simon was seen by a child psychiatrist and a speech therapist, who suggested to his parents that he might be a gifted child.

At the time of the first assessment, Simon was attending regular classes and did not receive special educational assistance. He had poor eye contact. His verbal communication initially seemed quite advanced, but he was pedantic in his choice of words. His voice was often too loud and his intonation theatrical. He often seemed to become lost in his own thoughts, and this had a considerable impact on his ability to hold conversations. Simon continually repeated sentences he had heard in cartoons. At school, he was a poor mixer, standing alone, and reading a book in the playground. Simon was unable to give his parents a chronological account of what he had done at school that day. For example, he might be able to tell his parents in incredible detail how he had fallen down in the schoolyard, but nothing else either before or after. He had no knowledge of social rules. He frequently committed faux pas in everyday life, such as asking people in the street if their dogs had fleas. It was very difficult to gain access to his own representations, feelings, emotions, and beliefs.

Simon had compulsive behavior, such as counting the steps as he went up or downstairs, or putting his things in his schoolbag in a particular manner. Simon was resistant to minor changes, such as switching between short-sleeved and long-sleeved clothes. He also displayed considerable restlessness, having difficulty sitting still in class.

Simon had a sensory/perceptual hypersensitivity. He was worried by sounds such as applause, a baby crying, a vacuum cleaner, or a hairdryer. He often put his hands over his ears, even when his parents could not hear anything. This auditory hypersensitivity was associated with acute attention to visuospatial details. By 4 years, he could already complete quite complex jigsaws. Simon was exceptionally good at construction toys. Whenever his father bought a piece of flat pack furniture, Simon was always the first to understand how to put it together.

Therapeutic interventions during these 3 years included psychiatric consultations, individual psychotherapy sessions, educational interventions, psychomotor therapy, and cognitive remediation. Cognitive remediation is a specific and individualized training program designed to reduce symptoms and improve cognitive functions and psychosocial functioning. It consists of exercises and practice of increasing difficulty, using a range of materials. This program of cognitive remediation took place twice a week over an 18 month period, and focused on a range of abilities thought to underlie social interaction, including emotion perception, ToM, and pragmatic conversational abilities. The program targeted the comprehension of complex social emotions, the attribution of mental states (intentions, beliefs, feelings), the learning, and comprehension of social rules, the estimation of the consequences of one’s own behavior, and the comprehension of humor, irony, metaphoric utterances, and indirect requests. Simon also joined a social skills training group for a year. Social skills training seeks to improve social functioning. It is a structured method designed to teach the social interaction skills needed to build and foster interpersonal relationships, and promote the maintenance and generalization of these skills in everyday life. Group-based training in social skills means that the group dynamics can be used to facilitate therapeutic interactions in the “here and now.” The program consisted of 20 sessions over 7 months and targeted the following social skills: recognizing non-verbal communication, identifying, and expressing emotions, introducing oneself, and listening to others, starting, maintaining, and ending a conversation, making demands, making, and receiving compliments and criticism, reacting to teasing, giving, and receiving presents. All the sessions followed the same sequence of activities: (1) joke of the day, (2) review of the previous week’s homework assignments, (3) introduction of a new skill, (4) practice (direct instruction, modeling, role play, etc.), (5) presentation of the new homework assignments, and (6) social play time.

### Neuropsychological examinations

*A general cognitive assessment* was conducted three times. It covered intellectual ability (Wechsler Intelligence Scale for Children, WISC-IV; Wechsler, 2005), attention, executive functions (Tower of London, Visual attention, Auditory attention, Response set, and Verbal fluency subtests of the Developmental Neuropsychological Assessment, NEPSY; Korkman, 2003; Stroop-Drawing; Pennequin et al., 2004), working memory (WISC-IV, Corsi blocks; Pagulayan et al., 2006), and episodic memory (Rivermead Behavioral Memory Test; Wilson et al., 2000).

*Theory of mind abilities* were assessed using a false-belief task (Desgranges et al., 2012). This task was based on false-belief cartoon tasks such as “Sally and Ann” (Wimmer and Perner, 1983). We used eight short comic strips illustrating first-order false-belief scenarios that had been developed within our laboratory. Each comic strip comprised three pictures with a short written description (for examples, see Bon et al., 2009; Desgranges et al., 2012). The aim was to understand the story by reading the scenario, then answer a question with two possible responses. There were two conditions. In the ToM condition, the question was about the belief of one of the characters in the story. In the control condition, the question probed Simon’s understanding of the reality of the cartoon scenario. The pictures and written descriptions remained visible throughout.

*Autobiographical memory* was assessed using an adaptation of the *Test Episodique de Mémoire du Passé autobiographique* (TEMPau task), a semi-structured autobiographical questionnaire validated in school-age children (Piolino et al., 2007; Picard et al., 2009). Briefly, this task explored personal information and specific events pertaining to three different lifetime periods (current school year, previous school year, and earlier school years).

For each period, personal information had to be recalled on four different topics: the names of acquaintances, personally relevant famous names (heroes, stars, etc.), information about school life, and personal addresses and regular activities. Specific instructions were given for each period (e.g., “Can you give me the names of three people from among your acquaintances during this period, such as the name of a friend, a neighbor, or a teacher?”). Simon was asked to supply this information as accurately as possible. In particular, he was asked to avoid giving information that was applicable to several periods and to provide information specific to the period under examination.

Concerning personal events, Simon was asked to recall specific, personally experienced events associated with specific dates for the current year period and prompted by three general topics (i.e., a school event, a trip, or vacation, and a family event) for the other two periods. For the current school year, six questions were used to conduct a chronological study of the previous months (last summer, last Christmas, last week, 2 days ago, yesterday, and today). Simon was asked to remember specifically experienced events that had lasted less than a day and which he could relive with details and situate in time and space (e.g., “Describe out loud and with as many details as possible, what happened, as if you were reliving it: what you did and felt, the circumstances, with whom, where, and how it happened”). Specific instructions were given for each theme, such as “Give details of a particular event that took place in your family life.” Simon was always asked to give as many details as he could. Where necessary, if he had difficulty carrying out the task, two types of help could be provided. If he failed to produce any recollections, he was prompted with cues (e.g., “A day with a teacher or a school friend”). If he produced a general memory, he was encouraged to be more specific about the spatiotemporal context and to describe the circumstances of a particular incident (“Does this remind you of a particular day?”, “Did this only happen once?”). The autobiographical questionnaire was provided to Simon’s parents before the test to collect specific information about his life and all the recollections he produced were checked with his parents after the test.

Scores were calculated separately for personal information and events, and for each period. For personal events, each event was scored on a four-point episodic scale, which took into account the specificity of the content (single or repeated event), the spatiotemporal context (place, date, and time), and the presence of specific details (perceptions, thoughts, images, emotions, etc.). An overall personal event score was obtained by adding up the points recorded per period, taking all types of recall into account, both specific and generic. For each period, the maximum score was 12 (four points *per* topic and three topics *per* period). Each accurately recalled item of personal information was scored half a point, in order to have a maximum score of 12 *per* period, to compare with personal event score.

Performances were compared with those of controls published in Piolino et al. (2007). Controls were typically developing volunteer children, recruited from elementary and junior high schools. None of them had any neurological or psychiatric medical history and repeated a year. For this study, Simon’s performances collected in 2007 and 2008 were compared with those of 14 controls aged from 7 to 8 (*M* = 8.14 years, SD = 0.77 years), and for the last examination conducted in 2010, performances were compared to those of 14 controls aged from 9 to 10 years old (*M* = 10.02 years, SD = 0.56 years). Two independent experts rated each child’s production: the tester who was different for the control group and Simon and a rater blind to the age of children who was the same for all children. In cases of disagreement, the data were re-examined until a consensus was reached.

## Results

The three general cognitive assessments yielded normal results for most of the tests (Table 1). Results on the WISC-IV indicated that Simon consistently performed within the average range, albeit with a significant decline in verbal comprehension, and superior perceptual abilities indicated by the Perceptual Reasoning Index. The neuropsychological examinations did not reveal any attention or executive disorders, and although we noted a significant impairment in the ability to generate words from a phonemic cue (NEPSY Verbal Fluency), Simon performed normally in the third examination. Verbal and visuospatial working memory was normal. Episodic memory, as measured with the Rivermead Behavioral Memory Test, was also normal. The second assessment of ToM with a first-order false-belief task (Desgranges et al., 2012) yielded pathological results, but Simon’s performance was normal in the third examination. For example, in the famous story “Maxi and his chocolate,” a young boy called Maxi puts some chocolates in a green cupboard. After Maxi has left the room, his mother moves the chocolate to a blue cupboard. Maxi returns and wants his chocolate (Wimmer and Perner, 1983). Simon was asked where Maxi would look for the chocolate. The first time, Simon answered that Maxi would look for his chocolate in the blue cupboard because “his mother put it here.” In the second examination, however, Simon concluded that Maxi would look for his chocolate in its original location, because “he doesn’t know his mother moved it,” adding “Nobody is telepathic!” Another story features a young boy who, because of his small stature, has to stand on a box to speak to his new neighbor over a high garden wall. Later on, his neighbor is in the street and thinks she recognizes the boy. The final picture in this comic strip shows two boys looking very similar, apart from their different sizes (a tall one and a short one). Simon was asked to say which one the neighbor would think she had chatted to. In 2008, Simon responded that it was the small boy, because “he is small and has to stand on a box.” In 2010, however, Simon gave the correct response (the tallest boy), adding that “She thinks he is tall because of the box.”

**Table 1:** **General cognitive assessment**.

Tasks		Simon’s scores		
		2007	2008	2010
WISC-IV	Verbal comprehension index	110	90	82
	Perceptual reasoning index	135	114	124
	Working memory index	109	103	112
	Processing speed index	88	83	86
Stroop-drawing	Control condition 1	47	68	73
	Control condition 2	55	64	64
	Interference condition	30	40	44
Verbal fluency (NEPSY)	Semantic criterion	8	23	26
	Phonemic criterion	3*	4*	14
Visual attention (NEPSY)	Precision (/40)	39	37	38
Auditory attention and response set (NEPSY)	Score (/132)	105	105	102
Tower of London (NEPSY)	Score (/20)	13	13	14
Knock and tap (NEPSY)	Score (/30)	26	26	29
Digit span	Forward	6	6	5
	Backward	3	4	5
	Corsi blocks	5	5	7
Rivermead behavioral memory test	Score (/22)	20	18	21
First-order false-belief	Score (/8)	/	3*	8

*The WISC-IV indices are shown in standard score format (*M* = 100 ± 15), whereas the other measures are shown as raw scores*.

*Indicates pathological scores.

**Figure 1:**
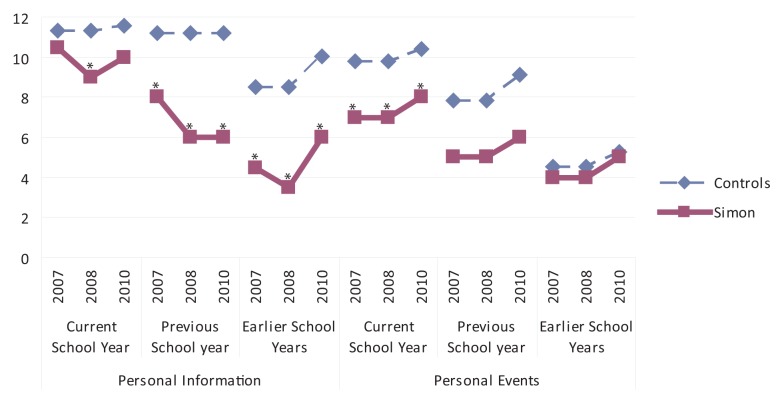
**Production of personal information and events across the three lifetime periods**. *p < 0.05. Legend: Simon’s scores were compared with those of controls matched on chronological age and published in Piolino et al. (2007). Simon obtained pathological scores (*Z* < 1.777; *p* < 0.05; df = 13) for personal information for the previous school year and earlier school years, as well as for the current school year, though only in 2008. Personal event scores were only pathological for the current school year period, although they were slightly low for the previous school year period (1.35 < *z* <1.77; 0.10 < *p* < 0.05).

Concerning AM, Simon exhibited a deficit in both components – personal information and personal events (Figure 1). For personal information, Simon performed just as well as controls for the current school year (except for the second assessment). However, for the previous year and earlier years, Simon performed below average in all three examinations.

Conversely, for personal events, at all three examinations, Simon exhibited pathological performances for the current year, below those of controls (though not significantly so) for the previous school year, and as good as those of controls for the earlier school years.

## Discussion

By distinguishing between events and personal information, and by looking at how these memories change in the course of development, our investigation of Simon’s AM revealed the relative preservation of personal knowledge for the current year, but impairment for the two previous periods. In terms of episodic AM development, Simon produced normal childhood memories, but his more recent ones were impaired.

### Semantic autobiographical memory

Bruck et al. (2007) also reported impairment of the semantic component of AM, but without the preservation of the most recent period. However, in their study, the authors assessed the current year using 23 questions that were far more specific than those administered in the present study (e.g., the names of the child’s doctor, teacher, principal; the age and occupation of his or her parents). This more detailed exploration may have been more sensitive to mild impairment than our task. Nonetheless, the pathological results regarding the two earlier periods suggest that Simon forgot a significant proportion of his personal or self-related information. In the 2010 examination, for example, concerning information about his current school, Simon knew the name of his teacher and could provide some details about his classroom, such as the location of his desk. For the previous school year, however, Simon was only able to give his teacher’s name, and for the earlier period, Simon was not able to produce any details associated with his school. The longitudinal approach we adopted proved highly informative. In 2007, Simon listed the names of three ferries he used to travel on when he lived in another country. By 2008, he had forgotten two of them and only produced the third one when the examiner prompted him with the first two names. In 2010, he was not able to cite a single name. Some personal information may be recalled with cues, but some may be completely forgotten, as attested by some false productions (e.g., in 2008, the name of the teacher he had had 2 years earlier). These results suggest that personal information fades, with retrieval difficulties first, followed by genuine, accelerated, long-term forgetting. There are two possible explanations, not necessarily mutually exclusive. First, this semantic pattern may be explained by an abnormal mechanism of consolidation *per se* that needs verbal communication and social interaction to be efficient. Limited linguistic interactions and personal thoughts in ASD prevent rehearsal which, in a typical context, strengthens semantic memories (Kopelman and Bright, 2012). Second, there may be a deficit in semanticization, or the episodic-to-semantic shift, which also relies on verbal communication and social interaction to turn episodic memories into semantic ones via a mechanism of repetition and updating (Cermak, 1984). The limited verbal interaction associated with the episodic memory deficit may also hinder the addition of new semantic information, not least encyclopedic knowledge, as demonstrated by the decrease in verbal comprehension assessed with the WISC-IV.

### Episodic autobiographical memory

As regards the episodic component of AM and in accordance with our predictions, results suggested a deficit in the production of personal events associated with objective (i.e., spatiotemporal context) and subjective phenomenological details, including thoughts and emotions. These results mirror previous findings of impaired episodic AM in high-functioning individuals. The representations of events held by individuals with ASD are more general and temporally non-specific (Crane and Goddard, 2008). They resemble the event memories produced by younger typically developing children before episodic memory has fully developed (Maister and Plaisted-Grant, 2011). If Simon’s performances were similar to those of controls for the earliest lifetime period (earlier school years), it is because personal event memory for this period is not yet truly episodic. Children with ASD are able to produce fragmented memories based mainly on perceptual images, sometimes with disconcertingly specific details, but these are not integrated into a temporal continuum (Newcombe et al., 2007). This developmental limitation explains why the ability to produce episodic autobiographical memories associated with distant lifetime periods does not improve when children grow up (Picard et al., 2009). Hence, the development of episodic memory may be limited to a primary level containing some specific details but without recollective abilities or the subjective experience of mental time travel. For example, in 2008, Simon recalled his birthday party, which had taken place 5 months earlier. He was able to list the people who had been at the party, where it was with some specific places, what he had eaten and some of the presents he had received, but all these details were produced as though he were describing a picture. When he was asked if he remembered that event and something he did there, he answered “no.” He had no subjective experience allowing him to recollect this personal event.

There are important interdependencies between episodic and semantic AM. First, the semantic impairment may contribute to the encoding and retrieval deficits in episodic memory. The acquisition of complex episodic memories relies on semantic information, while personal knowledge allows us to access specific sensory/perceptual episodic memories. Episodic AM and semantic AM are highly interconnected, especially during the early stages of retrieval when personal semantic knowledge can aid memory search and retrieval operations (Conway and Pleydell-Pearce, 2000; Svoboda et al., 2006). Moreover, semantic AM develops earlier and provides a foundation for the later and more gradual development of episodic AM in typically functioning children (Picard et al., 2009).

### Social cognition and AM

The follow-up showed an improvement in performances on the first-order false-belief task. This may have contributed to the improvement in Simon’s episodic AM by increasing his ability to understand that that which is brought to mind during the act of remembering refers to the mental state of a past experience (Perner, 2000; Naito, 2003; Perner et al., 2007). In young adults with Asperger’s syndrome or high-functioning autism, correlations have been found between performances on a test which assesses the affective ToM (the Reading the Mind in the Eyes Test) and performances on an AM task (Adler et al., 2010). Therapeutic interventions may have contributed to this relative improvement in episodic AM. These interventions encourage verbalization and narrative abilities, and may thus have affected Simon’s ability to recount personal experiences. However, this improvement may not be enough for children with ASD to reach a normal level of AM function. Furthermore, false-belief task performance may not always be a reliable indicator of representational ToM among these children, who may draw on other cognitive abilities to perform this task (Lind and Bowler, 2009a,b). Thus, on the basis of Simon’s results, we cannot entirely rule out the possibility of impaired ToM. Further investigations are needed to conclude on this point.

## Conclusion

This longitudinal approach yielded further explanations for the atypical functioning of AM in ASD. First, when we assessed personal information associated with specific and limited lifetime periods (e.g., previous year), we were able to confirm the impairment of the semantic component of AM that has previously been reported in children with ASD. This impairment may also be present in adulthood, but up to now, researchers have chosen to use questionnaires assessing longer lifetime periods than in the present study, covering more than 1 year (5 years for Crane and Goddard, 2008). Second, we also demonstrated an impairment of current and recent personal event memories, with the preservation of remote ones referring to event that occurred before the age of 7 years. These results suggest that patients with ASD may have a basic form of episodic memory resembling the event memory observed in children under six. Third, we observed an encouraging qualitative improvement in AM that may have resulted from a combination of different factors, including development, family support, and therapeutic interventions.

## Conflict of Interest Statement

The authors declare that the research was conducted in the absence of any commercial or financial relationships that could be construed as a potential conflict of interest.
